# Prevalence and Predictors of Nonalcoholic Steatohepatitis Using Multiple Non-Invasive Methods: Data from NHANES III

**Published:** 2020-12-30

**Authors:** Magda Shaheen, Katrina M Schrode, Dulcie Kermah, Deyu Pan, Vishwajeet Puri, Ali Zarrinpar, David Elisha, Sonia Michael Najjar, Theodore C Friedman

**Affiliations:** 1 Department of Internal Medicine, Charles R. Drew University of Medicine and Science, Los Angeles, USA;; 2 Department of Biomedical Sciences and Diabetes, Ohio University-Heritage College of Medicine, Athens, USA;; 3 Department of Surgery, University of Florida School of Medicine, Gainesville, USA

**Keywords:** Non-alcoholic fatty liver, Nonalcoholic steatohepatitis, Obesity, Non-invasive methods, NHANES III

## Abstract

**Objective:**

Patients with nonalcoholic steatohepatitis (NASH) are at risk for developing cirrhosis and hepatic cancer. Currently, the definitive gold-standard method of diagnosing NASH is a liver biopsy, an invasive and costly method. Our objective was to compare three non-invasive methods of identifying NASH by using data on 10,007 subjects from NHANES III (1988–1994) to determine the prevalence and variables associated with NASH, as defined by each non-invasive method.

**Methods:**

We used ultrasound data to identify subjects with moderate-to-severe hepatic steatosis, of whom we identified the NASH population using either the HAIR score, the NASH liver fat score, or the Gholam score, each of which had been validated with liver biopsy. We performed multinomial logistic regression to compare each NASH population to the normal population (those with no-to-mild hepatic steatosis).

**Results:**

We identified 1136 (9.5%) subjects as having NASH by at least one method and 219 (1.8%) were identified by all 3 methods. Independent of the non-invasive method used, Mexican-Americans (MA) had the highest prevalence of NASH. All three methods identified significant risk factors for NASH (p<0.05), including: elevated waist-to-hip ratio, elevated levels of C-peptide, total cholesterol, or C-reactive protein (CRP).

**Conclusion:**

We conclude that the combined non-invasive methods can help identify candidates with a high likelihood of being diagnosed with NASH. Health care providers can screen people with the combined non-invasive methods for the risk factors and identify candidates for interventions, including exercise and/or referral to biopsy.

## INTRODUCTION

Nonalcoholic steatohepatitis (NASH) is a serious liver condition marked by hepatic steatosis, steatohepatitis, cell injury and apoptosis. Hepatic steatosis is a characteristic of several liver diseases, including alcoholic fatty liver disease and non-alcoholic fatty liver (NAFLD). A subset of individuals with NAFLD develops NASH, which can progress to liver cirrhosis and cancer [1–3].

Several studies have suggested possible risk factors for NASH, such as insulin resistance [4] and Hispanic ethnicity [5]. Identification of such risk factors is best done in a population-based sample, because it requires healthy individuals for comparison. Currently, the most definitive method of diagnosing NASH is by liver biopsy, but since liver biopsy is invasive and costly, non-invasive methods of identifying NASH are desirable. Ample effort has been made to develop non-invasive methods of identifying NASH [6–9]; however, as yet, none of these methods has been accepted as a “gold standard.” These methods are often developed in specific populations, such as an obese population, potentially limiting their application in other groups. Few studies have verified the validity of a method in any population beyond the initial population used to develop non-invasive methods to diagnose NASH.

There have been several recent reviews of non-invasive methods for identifying NASH [6–9]. However, to our knowledge, no study has analyzed non-invasive methods both individually or in combination to identify NASH in a single, large population sample. Thus, we set forth to use three non-invasive methods to determine the prevalence of NASH and its predictors in a large representative sample of the US population. These include: the HAIR score [4], the Gholam score [10], and the NASH liver fat score [11], and we examined them either individually or combined, or at least one of these three methods to identify NASH and its associated factors. We used data from the National Health and Nutrition Examination Survey III (NHANES III) 1988–1994, because it is the only nationally representative sample of the US population that includes imaging data for identification of hepatic steatosis, as well as the data for the important potential predictors.

## METHODS

**Figure 1:**
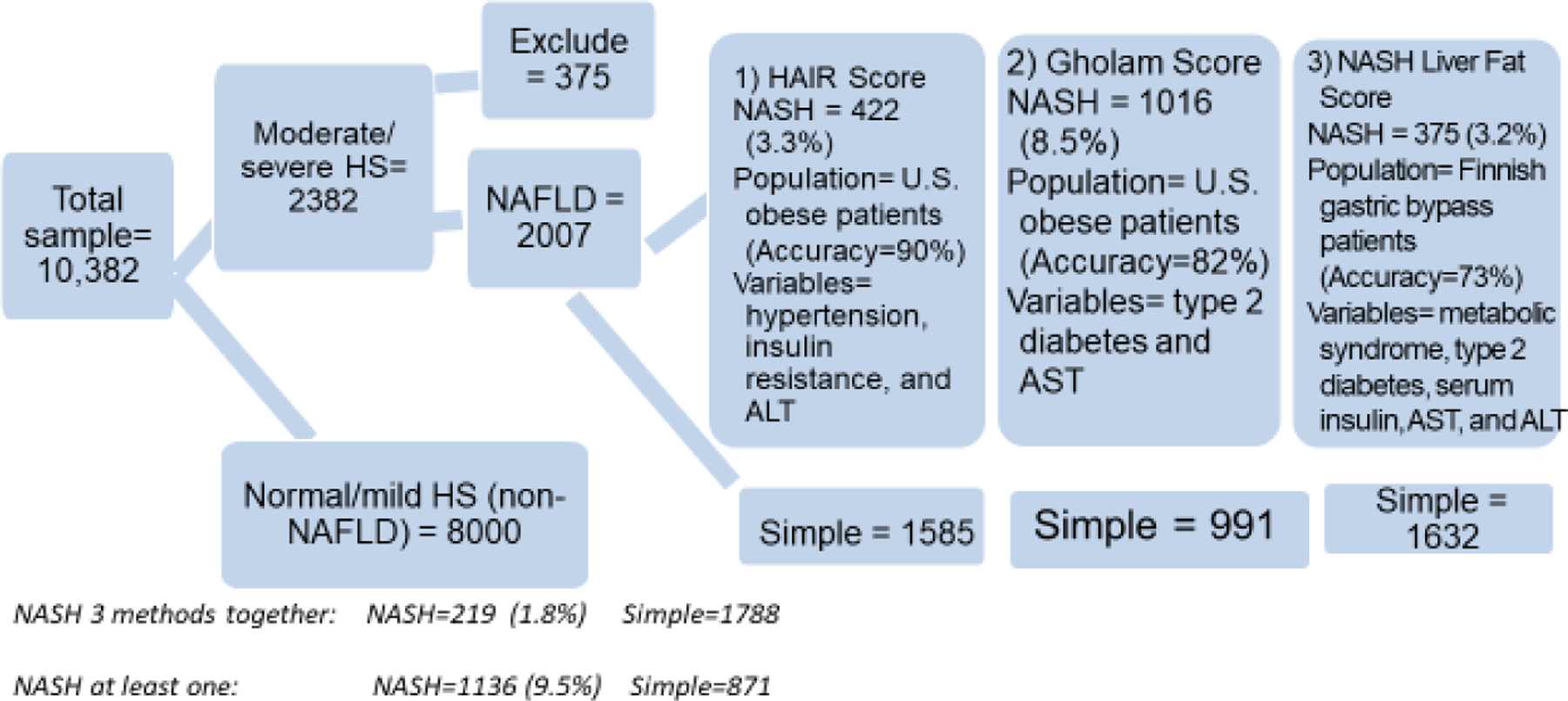
Flow diagram for the study sample from NHANES III used for analysis.

We analyzed data from the National Health and Nutrition Examination Survey III (NHANES III) 1988–1994 [12]. NHANES is a cross-sectional survey conducted by the National Center for Health Statistics, using a stratified multistage probability sample to obtain a representative sample of the total civilian, non-institutionalized U.S. population. The CDC had Institutional Review Board approval for NHANES, conformed to the ethical guidelines of the 1975 Declaration of Helsinki, and informed consent was obtained from all participants. Trained interviewers administered surveys in participants’ homes to obtain self-reported information. In addition, physical examinations and laboratory testing using blood and urine samples were conducted at mobile examination centers. The eligible population consisted of participants 20 to 74 years old (n=14,797). We excluded those with no hepatic ultrasound data or ungradable or inaccessible results (n=887). There was no missing race/ethnicity data. For the missing data for the other independent variables, we used the listwise deletion option in the analysis and also excluded data for 375 people with excessive alcohol intake, other liver disease (see below) and those taking drugs affecting the liver. This resulted in a total analytic sample of 10,007 (8,000 with normal/mild steatosis and 2,007 with NAFLD). We applied the non-invasive methods to the sample of 2,007 and we analyzed the data comparing normal/mild steatosis, simple NAFLD, and NASH groups. A flow diagram illustrating the sample used for analysis is shown in Figure 1.

### Liver measures:

Hepatic ultrasound data identified subjects with no, mild, moderate, and severe hepatic steatosis. To limit the population to NAFLD and no other causes of liver damage, subjects with elevated serum transferrin level >50% (an indicator of a predisposition for iron overload), chronic hepatitis B, chronic hepatitis C, excessive alcohol use, or taking prescription medications that known to cause hepatic steatosis were excluded. Chronic hepatitis B was defined as positive results for both the hepatitis B surface antigen (HBsAg: SAP) and hepatitis B core antibody (anti-HBc: HBP) tests. Chronic hepatitis C was defined as positive results for both the hepatitis C antibody (HCV: HCP) and RNA tests (HCPRNA). Average alcohol use was determined by multiplying the response to each of the two questions: “number of days drank alcohol in past 12 months” and “average drinks on drinking day” and dividing by 365 to get a daily average [13]. Excessive alcohol use was defined as an average of more than two drinks/day for men or more than one drink/day for women.

From the NAFLD population (N=2,007), we used three different non-invasive methods to identify the NASH population. These three methods are: the HAIR score [4], the Gholam score [10], and the NASH liver fat score [11]. The HAIR score was developed in a sample of obese patients and was defined by three components.

### Components:

HOMA insulin resistance (HOMA-IR), hypertension, and alanine aminotransferase (ALT). Subjects received 1 point for each pathological value, for a possible total of 3. The cutoffs used for each variable were HOMA-IR>5, ALT>40, and blood pressure ≥140/90 or prior diagnosis of hypertension or prescription of hypertension medication. Subjects were considered to have NASH if they have NAFLD and a HAIR score of ≥ 2. The HAIR score had an Area Under the Receiver Operating Curve (AUROC) of 0.9, a sensitivity of 0.8, and a specificity of 0.89 [4]. The Gholam score was developed in a sample of obese patients and used aspartate aminotransferase (AST) and a type 2 diabetes diagnosis. The score was calculated using the formula 2.627 *ln AST (+2.13 for patients with type 2 diabetes [prior doctor diagnosis or fasting glucose >126 mg/dL]). A subject was considered to have NASH if the score was ≥ 8.22. The Gholam score had an AUROC of 0.82, a sensitivity of 0.76, and a specificity of 0.66 (10). The NASH liver fat score was developed in a Finnish population undergoing gastric bypass, and validated in an Italian population of liver biopsy patients. The cutoff to determine if a subject had NASH was a score of 2.122. This score was calculated using the formula 1.18* metabolic syndrome + 0.45* type 2 diabetes +0.15* serum insulin + 0.04* AST − 0.94* (AST/ALT)-2.89, where metabolic syndrome is coded 0=no; 1=yes and diabetes is coded 0=no; 2=yes. Metabolic syndrome was defined as having at least 3 of the following criteria: waist circumference >94 cm in men; >80 cm in women; fasting triglyceride ≥ 150 mg/dL; HDL <40 mg/dL in men; <50 mg/dL in women; blood pressure ≥130/85 mmHg or being treated for hypertension; fasting glucose ≥ 100 mg/dL or being treated for hyperglycemia. Diabetes was defined as prior doctor diagnosis or fasting glucose ≥ 126 mg/dL or 2-hr glucose ≥ 200 mg/dL. In the Finnish and Italian populations, the NASH liver fat scores had AUROCs of 0.73 and 0.74, sensitivities of 59.5 and 92.9, and specificities of 79.7 and 32.7 [11], respectively. Subjects were classified using each non-invasive method as normal (no-to-mild hepatic steatosis), simple NAFLD and NASH.

In addition to evaluating each method individually (method 1), we also identified NASH in two additional ways, by combining the three methods and by at least one method. For the former, we estimated the net sensitivity as follows: if method 1 and method 2 are positive, then sensitivity [SE]=SE method 1* SE method 2 and Specificity [SP]=SP method 1+SP method 2-(SP method 1 * SP method 2). The resulting net sensitivity (range=36%–56%) is less than that of any one method, but the net specificity (range= 97–99) is higher than any single method [14].

To evaluate NASH by at least one method, we estimated the net sensitivity using the following: if method 1 or method 2 is positive, then Sensitivity [SE]=SE method 1+SE method 2-(SE method 1*SE method 2) and Specificity [SP]=SP method 1* SP method 2. The resulting net sensitivity (range=97%–98%) is higher than that of any one method but the net specificity (range=19%–47%) will be lower than any single method.

### Independent variables and measures:

The following variables were included in the analyses: demographics (age, gender, education, urbanization, and poverty), physical activity status, smoking status, body composition (waist circumference and waist-hip ratio), laboratory values [triglyceride, cholesterol, glucose, HbA1c, HOMA-IR, and C-reactive protein (CRP), ALT and AST], and healthy-eating index (HEI). However, variables that were part of a NASH definition were not included in the regression analysis.

Physical activity was classified into 3 categories based on the frequency of activity per week (0=Inactive; 1=not meeting guidelines, i.e., moderate exercise of <5 times/week or vigorous exercise <3 times/week; and 2= meeting guidelines), based on recommendations from the American College of Sports Medicine at the time of the survey [15–17]. Age was categorized as 20–34 years; 35–49 years;] 50 years and older. Education was categorized as less than high-school (<12th grade), high-school (12th grade), and more than high-school (>12th grade). Gender was categorized as male or female.

Race/ethnicity was categorized as White, non-Hispanic Black (Black), Mexican-American (MA) or others. Federal poverty level (FPL) was classified as <1, 1–2, and >2 FPL. Urbanization classification was based on USDA Rural/Urban continuum codes. (1= central counties of metro areas of ≥ 1 million population or fringe counties of metro areas of ≥1 million population; 2=all other areas [rural]).

Alcohol status was classified as current, former, and did not drink alcohol based on the response to the following questions: “In your entire life, have you had at least 12 drinks of any kind of alcoholic beverage and “In the past 12 months did you have at least 12 drinks of any kind of alcoholic beverage” If the response is “Yes” to the first question, then a subject is classified as current drinker. If the response is “Yes” to the first question and “No” to the second question, then subject is classified as former drinker. If the response is “No” to the first question, then the subject is classified as did not drink alcohol.

Smoking status was categorized into a non-smoker, former and current smokers. Waist-to-hip ratio was categorized as normal (<0.85 for women, <0.9 for men), or high risk (>0.85 as for women and >0.9 for men).

C-peptide levels were classified as low (<0.26 nmol/L), normal (0.26–1.03 nmol/L), and high (>1.03 nmol/L). Total cholesterol was categorized as normal ( ≤ 200 mg/dL), elevated (200–239 mg/dL), and high (≥240 mg/dL). CRP was categorized as normal (0.1–0.3 mg/dL), mild (mild inflammation) (0.3–1 mg/dL), and high (significant inflammation) (>1 mg/dL). Based on the HEI total score, subjects were classified into having poor diet (HEI score <50), diet needed improvement (HEI score=50–80) and good diet (HEI score=80–100).

### Statistical analyses:

Descriptive statistics were used to characterize the study population. We report un-weighted number and weighted percent for the categorical variables.

Age-adjusted prevalence estimates of NASH were calculated by using direct standardization with age group proportions based on 2000 US Census data. We used these age-group proportions to weight the estimates of the NASH prevalence identified by each individual method, identified by at least one method, and identified by all methods combined [18].

Bivariate analysis using the chi-Squared test for categorical variables was used to determine the statistical difference between the prevalence of NASH in racial/ethnic groups and in the other population characteristics.

Multinomial logistic regression was used to compare the NASH population to the normal population (those with no or mild hepatic steatosis) in the predictors of NASH. We developed a model for each non-invasive method of diagnosis separately. In addition, we developed a model for the prevalence of NASH by the three combined methods and by at least one method. Results are presented as unadjusted and adjusted odds ratio with 95% confidence interval. A p-value of <0.05 was considered statistically significant. Data were analyzed using SAS (Release V.9.1.3, 2002; SAS, Inc) and the survey module of STATA version 14. Sample weights, provided by the NCHS, were used to correct differential selection probabilities and adjust for non-coverage and non-response [19]. All estimates were weighted using weights supplied by NHANES and taking the multistage cluster sample design into consideration [19].

## RESULTS

Of the 10,007 subjects in our sample from NHANES 1988–1994, 37.1% were 20–34 years old and 10.8% were 65 years and older. 10.2% were Black and 4.3% were Mexican-American (MA). About half of the population were male (47.9%), 53.6% lived in rural areas, 20.8% had less than high-school education and 11.6% were poor (federal poverty level <1). 30.2% were current smokers, 14.0% were physically inactive (did no exercise), 41.6% did not meet the exercise guidelines, 65.1% had high waist-to-hip ratio ( ≥ 0.85 for males, ≥ 0.9 for females) and 16.2% had poor diet quality based on HEI score. About nineteen percent (18.6%) had high total cholesterol ( ≥ 240 mg/dL), 6.3% had significant inflammation as indicated by >1 mg/dL CRP level, and 17.4% had high C-peptide level (Table 1).

The overall prevalence of NASH was 3.3% using the HAIR score, 8.5% using the Gholam score, and 3.2% using the NASH Liver Fat score separately. When subjects were positive by all of the methods combined, NASH prevalence was 1.9%, and when subjects were positive by at least one method, the prevalence of NASH was 9.5%.

The age adjusted prevalence of NASH using the direct adjustment method (1990 census standard population) was 4.2% by the HAIR score, 10.0% by the Gholam score, and 3.7% by the NASH Liver Fat score. Using the three methods combined, the age-adjusted prevalence was 2.2% and for diagnosing NASH using at least one positive method, the age-adjusted prevalence was 11.2%.

The prevalence of NASH was highest among subjects of 50–64 years of age by all three methods (p<0.05): for HAIR Score (6.1%), Gholam Score (12.2%), NASH Liver Fat Score (3.9%), (3.1%) when three methods were combined and 13.5% when at least one method was used.

Independent of the non-invasive method used, Mexican-Americans had the highest prevalence of NASH and, except when using the HAIR score, non-Hispanic Blacks had the lowest prevalence of NASH. Using the HAIR Score, NASH had a prevalence of 5.3% among Mexican-Americans, 3.2% among Whites and 3.3% among Blacks. Using the Gholam Score, NASH had a prevalence of 14.7% among Mexican-Americans and 7.2% among Blacks. Using NASH liver fat score, NASH had a prevalence of 5.6% among Mexican-Americans, and 2.6% among Blacks. Combining the 3 methods, NASH had a prevalence of 3.5% among Mexican-Americans and 1.4% among Blacks, and using at least 1 method, NASH had a prevalence of 15.7% among Mexican-Americans and 8.5% among Blacks. The differences in prevalence were statistically significant (p<0.05), irrespective of the non-invasive method used (Table 1).

NASH prevalence was the highest among subjects with high inflammation (as indicated by CRP level >1 mg/dL) based on all methods except for when Gholam was used individually, NASH appeared to be highest among those with mild inflammation (0.3–1 mg/dL) (p<0.05). Males, subjects who lived in rural areas, those with less than high-school education, ex-smokers, physically inactive and those with high waist-to-hip ratio, C-peptide and total cholesterol levels had the highest prevalence of NASH using all the non-invasive methods (p<0.05) (Table 1).

### Factors associated with NASH comparing the three non-invasive methods

Multivariable regressions adjusting for other covariates showed independent statistical associations between NASH (as diagnosed individually by HAIR Score, Gholam Score, or NASH Liver Fat Score) and race/ethnicity [Mexican-Americans versus Whites], waist-to-hip ratio [high versus healthy], smoking status [current versus never], C-peptide [high versus normal], total cholesterol [high versus normal], and CRP levels [mild or significant versus normal] (p<0.05) (Table 2). Gender [female versus male] was independently associated with NASH using the Gholam score (p<0.05). Location (rural versus urban) was independently associated with NASH using either the HAIR Score or the NASH Liver Fat Score methods (p<0.05). Country of birth and length of stay in the US were independently associated with NASH using HAIR Score and NASH Liver Fat Score (p<0.05). The HEI was not associated with NASH using any method. Physical activity was independently associated with NASH using only the Liver Fat Score method where those who did not meet guidelines had a higher chance of developing NASH compared to those who met guidelines (AOR=1.6, 95% CI=1.2–2.2, p<0.05) (Table 2).

### Factors associated with NASH diagnosis when all combined scores were positive

Independent significant risk factors that were associated with NASH diagnosis were race/ethnicity (other groups relative to Whites) (AOR=7.2, 95% CI=1.9–27.6, p<0.05), high waist-to-hip ratio versus normal (AOR=7.5, 95% CI=2.6–21.6, p<0.05), former alcohol drinkers versus those who did not drink alcohol (AOR=2.3, 95% CI=1.1–4.7, p<0.05) , higher than normal level of C-peptide (AOR=40.9, 95% CI=15.7–106.0, p<0.05) and of cholesterol (AOR=2.2, 95% CI=1.1–4.4, p<0.05), mild inflammation [CRP of 0.3–1.0 mg/dL] (AOR=1.9, 95% CI=1.2–3.1, p<0.05) and significantly higher than normal inflammation [CRP>1 mg/dL] (AOR=2.1, 95% CI=1.2–3.8, p<0.05). There was no association between current alcohol drinking status versus those who did not drink alcohol and NASH.

### Factors associated with NASH using at least one non-invasive method of diagnosis

Independent significant risk factors for NASH identified by at least one method of non-invasive diagnosis were race/ethnicity (Mexican-Americans versus Whites [AOR=1.6, 95% CI=1.2–2.0, p<0.05]), high versus normal waist-to-hip ratio [females ≥0.85, males ≥0.9] (AOR=3.0, 95% CI=2.1–4.4, p<0.05), physical activity where those who did not meet exercise guidelines had higher chance to develop NASH compared to those who met guidelines (AOR=1.3, 95% CI=1.01–1.6, p<0.05), and elevated levels of C-peptide [>1.03 nmol/L] (AOR=6.6, 95% CI=5.1–8.5, p<0.05) and cholesterol [200–239 mg/dL] (AOR=1.4, 95% CI=1.1–1.8, p<0.05) relative to normal, higher than normal cholesterol [≥240 mg/dL] (AOR=1.4, 95% CI=1.11.9, p<0.05), as well as mild [CRP of 0.3–1.0 mg/dL] (AOR=1.7, 95% CI=1.3–2.2, p<0.05), and significant inflammation [CRP>1 mg/dL] (AOR=1.5, 95% CI=1.0–2.0, p<0.05) compared to normal (Table 2).

## DISCUSSION

Our study used three non-invasive methods (HAIR score, Gholam score, and NASH Fat Liver Score) to identify the prevalence and risk factors independently associated with the likelihood of NASH diagnosis in the representative sample of the non-institutionalized US population who had hepatic ultrasound data in NHANES III. Our results were based on each non-invasive method separately (i.e., the HAIR score, had an AUROC, sensitivity, and specificity of 0.9, 80%, and 89%, respectively) and in combination (i.e., positive by at least one non-invasive method where the range of net sensitivity is 97% to 99%). Several other non-invasive methods have been used to identify NASH. Most of these used clinical or biochemical markers, and have accuracy similar to the methods we tested, but lower sensitivity compared to the combined methods we analyzed with high net sensitivity. For example, Campos *et al.,* [20] developed a clinical scoring system with an AUROC of 0.8; Palekar *et al.,* [21] developed a model based on clinical markers with an AUROC, sensitivity, and specificity of 0.76, 73.7%, and 65.7%, respectively. The NashTest biochemical panel showed similar accuracy with an AUROC of 0.79 and 94% specificity, but the sensitivity only reached 33% [22]. The NASH Risk Score [23] combined several clinical and biomedical markers and had an AUROC of 0.76. In another study, after developing and validating NASH score and NASH Liver Fat Score in smaller cohorts, Hyysalo *et al.,* [11] calculated the prevalence of NASH in a stratified sample of ^~^3000 Finnish subjects by the NASH Score and NASH Liver Fat Score as 6.0% and 4.2%, respectively. The novelty of the current study was that we tested these three methods in a large population sample and estimated a high net sensitivity.

In our study, we estimated the age-adjusted prevalence of NASH using HAIR Score as 4.2% and by NASH Liver Fat Score as 3.7%, by Gholam Score as 10.0%, and by at least one score as 11.2%. Estimates report 3–5% of adults in the US had NASH in 1988–1994 through a modeled approximation that assumes that 20% of NAFLD cases would be classified as NASH [24]. This estimate was close to our estimates using HAIR Score and NASH Liver Fat Score, higher than using the three combined score, but lower than our estimate using the criteria of diagnosing NASH by at least any one score. The net sensitivity was highest for the diagnosis by at least any one score and the net specificity was highest using the combined three scores. Kim *et al.,* [25] and Williams *et al.,* [26] reported a NASH prevalence of 12% based on non-invasive fibrosis markers, ultrasound and biopsy among the largely middle-aged population. This prevalence was close to our estimate of the age-adjusted prevalence using the diagnosis by at least any one method [25, 26]. Most experts expect the rate of both NAFLD and NASH to increase dramatically leading to increased cirrhosis and hepatocellular carcinoma with resultant increases in liver transplantation [27].

In the multivariable analysis, we found that independent of the method of diagnosis, people more likely to be diagnosed with NASH were Mexican-Americans [26], male [5], and those with high waist-to-hip ratio [28], high levels of C-peptide [29], cholesterol [30], and CRP [31]. These findings are consistent with the previous investigations indicating the association of these factors with an increased likelihood of NAFLD and NASH [5,26,29–31]. Racial-ethnic differences in NASH may result from differences in the distribution of adiposity or differences in triglycerides because Hispanics have relatively more visceral adipose tissue and higher triglycerides than Blacks [32]. Hispanic women had a greater total adiposity than white women, which resulted from a higher fat percentage and fat mass in the trunk [33].

Surprisingly, although the unadjusted prevalence of NASH in our study was high among those with diet quality of needed improvement, as indicated by the HEI, the adjusted odds ratio of the association between diet quality and NASH was not statistically significant. This is a finding that needs further explanation as lifestyle modifications constitute primary therapy for the management of NAFLD and NASH [34]. This discrepancy could be due to measurement related issues in the estimation of the food intake by self-report or the cross-sectional nature of the study.

In addition, the diagnosis of NASH by all of the methods was independently associated with smoking status, where smokers were significantly less likely to be diagnosed with NASH relative to non-smokers, which warrants further investigation. A recent prospective cohort study by Ma et *al.,* indicated that higher adherence to healthy diets and physical activity, as well as smoking were associated with a lower risk of NAFLD [35]. Lazo and colleagues investigated the prevalence of hepatic steatosis and NAFLD and its predictors in the US population and found that prevalence was lowest among current smokers [36], consistent with our findings. However, our finding related to smoking is not consistent with other previous research showing smoking as a risk factor for NASH; this may be due to population differences or different methods of data collection or measurements.

We found higher odds for NASH diagnosis (using all the combined scores) in the former alcohol drinkers versus those who did not drink alcohol. There was no association between current alcohol drinking status versus those who did not drink alcohol and diagnosis of NASH.

Previous research regarding the relationship between alcohol consumption and NASH is controversial. Dunn *et al.,* [37] found in a cross-sectional study of 252 lifetime non-drinkers and 331 modest drinkers (i.e., modest drinkers is defined as up to 10 g of alcohol/day) that modest drinkers compared to nondrinkers had lower odds of having a diagnosis of NASH and the odds of NASH decreased with the increased frequency of alcohol consumption within the range of modest consumption.

Ajmera *et al.,* studied a population of 285 participants with NAFLD not receiving pharmacologic therapy of whom 168 were modest alcohol users (defined as ≤ 2 alcoholic drinks/day) and 117 were abstinent [38]. Comparing baseline and follow-up liver biopsies, they found that modest alcohol use was associated with lower odds of NASH resolution, compared to abstinence.

In our study, not meeting the physical activity guidelines was associated with an increased likelihood of NASH using at least one method which is consistent with previous research indicating that physical inactivity and the associated lower cardiorespiratory fitness have been related to an increase in NASH and its severity [39]. This is consistent with studies showing that diet, exercise and physical activity could be used in the management and prevention of NAFLD and NASH [34]. While Reha *et al.,* [40] found that factors like hypertension and diabetes did not predict NASH, studies by Campos *et al.,* [20] identified hypertension, diabetes, and high levels of AST and ALT as predictors of NASH. They, as well as Ulitsky *et al.,* [23] found sleep apnea to be a significant predictor of NASH [20]. We were unable to test sleep apnea as a predictor of NASH because of the limited number of participants diagnosed with sleep apnea in NHANES III.

Our current study has the strength of testing three non-invasive methods in a large population sample and estimation of net sensitivity and net specificity of the combined methods. Our study is limited, however, by the analysis of secondary data where some variables thought to be related to NAFLD/NASH were not available. We used data from NHANES III (1988–1994) that were collected around 30 years ago and it is likely that the demographics of NASH has changed in the years since these data were obtained; however, NHANES III data is the only representative sample of the US population with ultrasonography data, demographic data, behavioral and laboratory data. Some variables were measured by self-report (income, alcohol use, smoking history) which could be associated with recall bias. Since NHANES data is a cross-sectional study, we cannot infer a causal relationship or temporality, but we can measure association. Although we controlled for major confounders and robust associations, it is possible that other unknown confounders could account for the associations found. In addition, our study is limited by the small sample size in the subgroup analysis. We used equations that were developed using liver biopsies in other populations, but liver biopsies were not performed in our sample.

## CONCLUSION

We conclude that non-invasive methods can help identify the high-risk population and risk factors associated with NASH. Several common risk factors were identified by the combined three methods in the NHANES population. Care must be taken in classifying people with NASH based on one non-invasive methods because results differ depending on the method used, but using the combined methods improves the ability to identify the population at high likelihood of developing NASH. Health care providers can screen people with the combined non-invasive methods for the risk factors and identify candidates for referral to biopsy and/or could benefit from interventions including exercise. We plan to use the recently released 2017–2018 NHANES database that provides liver ultrasound elasticity measures to update the ability of the three non-invasive method scores to predict NASH diagnosis and its associated risk factors.

## Figures and Tables

**Table 1:** Sociodemographic characteristics of those with NASH as identified through 3 non-invasive methods.

			HAIR Score		Gholam Score		NASH Liver Fat Score		All 3 methods		At least 1 method	
	N (%)	N (%)	N (%)		N (%)		N (%)		N (%)		N (%)	
	Total (%)	Normal-to-mild Hepatic Steatosis	NASH	Simple	NASH	Simple	NASH	Simple	NASH	Simple	NASH	Simple
**Age (years)** *												
20–34	3551 (37.1)	3113 (89.1)	57 ( 1.3)	381 ( 9.6)	206 ( 4.9)	232 ( 6.0)	69 ( 1.8)	369 ( 9.1)	38 ( 1.0)	400 ( 9.9)	226 ( 5.6)	212 ( 5.3)
35–49	2889 (33.3)	2284 (82.3)	114 ( 3.0)	491 (14.6)	303 ( 8.2)	302 ( 9.4)	116 ( 3.5)	489 (14.2)	57 ( 1.5)	548 (16.1)	347 ( 9.5)	258 ( 8.2)
50–64	2108 (18.9)	1533 (73.7)	150 ( 6.1)	425 (20.1)	306 (13.6)	269 (12.6)	118 ( 5.1)	457 (21.2)	73 ( 3.1)	502 (23.1)	340 (15.0)	235 (11.2)
65+	1459 (10.8)	1070 (74.0)	101 ( 6.1)	288 (19.9)	201 (12.2)	188 (13.7)	72 ( 3.9)	317 (22.0)	51 ( 3.1)	338 (22.9)	223 (13.5)	166 (12.5)
**Race/Ethnicity** *												
White	4122 (79.8)	3323 (82.6)	159 ( 3.2)	640 (14.2)	374 ( 8.3)	425 ( 9.2)	128 ( 3.0)	671 (14.4)	81 ( 1.7)	718 (15.7)	415 ( 9.3)	384 ( 8.2)
Black	2969 (10.2)	2495 (84.6)	105 ( 3.3)	369 (12.1)	215 ( 7.2)	259 ( 8.2)	83 ( 2.6)	391 (12.7)	41 ( 1.4)	433 (14.0)	259 ( 8.5)	215 ( 6.9)
Mexican-American	2586 ( 4.3)	1918 (75.6)	148 ( 5.3)	520 (19.1)	397 (14.7)	271 ( 9.6)	150 ( 5.6)	518 (18.8)	89 ( 3.5)	579 (20.9)	428 (15.7)	240 ( 8.7)
Other	330 ( 5.6)	264 (79.6)	10 ( 3.3)	56 (17.1)	30 ( 8.8)	36 (11.6)	14 ( 4.9)	52 (15.5)	8 ( 2.7)	58 (17.6)	34 (10.9)	32 ( 9.5)
**Sex** *												
Male	4595 (47.9)	3599 (80.5)	206 ( 3.7)	790 (15.8)	588 (10.7)	408 ( 8.8)	174 ( 3.3)	822 (16.2)	101 ( 1.9)	895 (17.6)	636 (11.7)	360 ( 7.8)
Female	5412 (52.1)	4401 (84.0)	216 ( 3.0)	795 (13.0)	428 ( 6.4)	583 ( 9.7)	201 ( 3.2)	810 (12.9)	118 ( 1.7)	893 (14.3)	500 ( 7.6)	511 ( 8.5)
**Urban/rural** *												
Urban	4829 (46.4)	3951 (84.3)	159 ( 2.4)	719 (13.3)	452 ( 7.8)	426 ( 7.9)	154 ( 2.4)	724 (13.3)	79 ( 1.2)	799 (14.5)	502 ( 8.7)	376 ( 7.0)
Rural	5178 (53.6)	4049 (80.6)	263 ( 4.1)	866 (15.3)	564 ( 9.1)	565 (10.4)	221 ( 3.9)	908 (15.5)	140 ( 2.3)	989 (17.1)	634 (10.3)	495 ( 9.1)
**Education level** *												
Less than high school	3439 (20.8)	2578 (77.4)	211 ( 5.4)	650 (17.2)	446 (10.5)	415 (12.1)	175 ( 4.4)	686 (18.2)	107 ( 3.1)	754 (19.5)	506 (12.0)	355 (10.6)
High school	3389 (35.7)	2753 (81.1)	117 ( 3.5)	519 (15.5)	304 ( 8.3)	332 (10.7)	113 ( 3.8)	523 (15.2)	56 ( 1.6)	580 (17.3)	345 ( 9.8)	291 ( 9.2)
More than high school	3179 (43.5)	2669 (85.7)	94 ( 2.2)	416 (12.1)	266 ( 7.7)	244 ( 6.6)	87 ( 2.2)	423 (12.1)	56 ( 1.3)	454 (13.0)	285 ( 8.2)	225 ( 6.1)
**Federal poverty ratio**												
<1	2217 (11.6)	1727 (80.3)	111 ( 3.9)	379 (15.9)	253 ( 9.6)	237 (10.1)	112 ( 4.2)	378 (15.5)	62 ( 2.1)	428 (17.7)	288 (11.0)	202 ( 8.7)
1–2	2611 (19.9)	2075 (81.7)	121 ( 4.1)	415 (14.2)	269 ( 8.5)	267 ( 9.8)	99 ( 3.5)	437 (14.8)	56 ( 2.3)	480 (16.0)	305 ( 9.6)	231 ( 8.7)
>2	5179 (68.5)	4198 (82.8)	190 ( 3.0)	791 (14.2)	494 ( 8.2)	487 ( 8.9)	164 ( 3.0)	817 (14.2)	101 ( 1.6)	880 (15.6)	543 ( 9.3)	438 ( 7.9)
**Where born** ^a^												
United States	8522 (91.4)	6822 (82.3)	369 ( 3.4)	1331 (14.2)	843 ( 8.4)	857 ( 9.2)	302 ( 3.2)	1398 (14.5)	184 ( 1.8)	1516 (15.8)	946 ( 9.5)	754 ( 8.2)
Mexico	1002 ( 1.9)	766 (77.6)	47 ( 4.6)	189 (17.8)	143 (13.4)	93 ( 9.0)	63 ( 6.0)	173 (16.4)	32 ( 3.3)	204 (19.1)	155 (14.4)	81 ( 8.0)
Other	483 ( 6.7)	412 (83.7)	6 ( 1.1)	65 (15.3)	30 ( 7.3)	41 ( 9.1)	10 ( 2.5)	61 (13.9)	3 ( 0.7)	68 (15.7)	35 ( 9.0)	36 ( 7.4)
**How long lived in US**												
Entire life	8522 (91.4)	6822 (82.3)	369 ( 3.4)	1331 (14.2)	843 ( 8.4)	857 ( 9.2)	302 ( 3.2)	1398 (14.5)	184 ( 1.8)	1516 (15.8)	946 ( 9.5)	754 ( 8.2)
Less than 5 years	300 ( 1.5)	261 (84.9)	6 ( 1.1)	33 (14.1)	21 ( 9.7)	18 ( 5.4)	8 ( 1.4)	31 (13.8)	4 ( 0.8)	35 (14.3)	22 ( 9.8)	17 ( 5.3)
5–10 years	253 ( 1.5)	209 (88.9)	3 ( 0.4)	41 (10.7)	22 ( 4.9)	22 ( 6.2)	8 ( 4.5)	36 ( 6.6)	2 ( 0.2)	42 (10.9)	25 ( 8.4)	19 ( 2.7)
More than 10 years	932 ( 5.6)	708 (79.9)	44 ( 2.5)	180 (17.7)	130 ( 9.3)	94 (10.8)	57 ( 3.4)	167 (16.7)	29 ( 1.7)	195 (18.5)	143 (10.7)	81 ( 9.4)
**Waist-Hip Ratio** *												
Healthy	3058 (34.9)	2794 (92.7)	12 ( 0.3)	252 ( 6.9)	84 ( 2.0)	180 ( 5.3)	16 ( 0.5)	248 ( 6.8)	7 ( 0.1)	257 ( 7.1)	88 ( 2.2)	176 ( 5.0)
Risk for women (>= 0.85)/risk for men (>=0.9)	6949 (65.1)	5206 (76.7)	410 ( 4.9)	1333 (18.4)	932 (11.9)	811 (11.3)	359 ( 4.7)	1384 (18.6)	212 ( 2.7)	1531 (20.6)	1048 (13.4)	695 ( 9.8)
**Smoking status** *												
Current	2826 (30.2)	2392 (86.7)	60 ( 1.2)	374 (12.1)	190 ( 4.6)	244 ( 8.7)	55 ( 1.7)	379 (11.7)	24 ( 0.6)	410 (12.8)	213 ( 5.3)	221 ( 8.0)
Former	2368 (25.4)	1748 (75.8)	161 ( 6.6)	459 (17.6)	352 (12.5)	268 (11.8)	137 ( 5.7)	483 (18.6)	86 ( 3.5)	534 (20.8)	391 (14.5)	229 ( 9.8)
Never	4813 (44.4)	3860 (83.1)	201 ( 2.9)	752 (14.0)	474 ( 8.8)	479 ( 8.1)	183 ( 2.9)	770 (14.0)	109 ( 1.7)	844 (15.2)	532 ( 9.6)	421 ( 7.3)
**Alcohol use** *												
Current Drinkers	4956 (57.5)	4084 (85.3)	149 ( 2.2)	723 (12.5)	456 ( 7.2)	416 ( 7.6)	136 ( 2.2)	736 (12.5)	80 ( 1.2)	792 (13.5)	494 ( 7.8)	378 ( 6.9)
Historical drinkers	3553 (31.6)	2740 (78.1)	203 ( 5.3)	610 (16.7)	414 (10.4)	399 (11.6)	179 ( 4.9)	634 (17.0)	107 ( 2.9)	706 (19.0)	470 (12.2)	343 ( 9.8)
Never drinkers	1498 (10.9)	1176 (79.2)	70 ( 3.4)	252 (17.4)	146 ( 9.6)	176 (11.2)	60 ( 3.5)	262 (17.4)	32 ( 1.5)	290 (19.3)	172 (11.1)	150 ( 9.7)
**Healthy eating index**												
Poor diet	1782 (16.2)	1443 (82.6)	74 ( 2.8)	265 (14.6)	167 ( 6.9)	172 (10.5)	52 ( 2.1)	287 (15.3)	32 ( 1.5)	307 (15.9)	186 ( 7.6)	153 ( 9.8)
Needs improvement	7239 (73.1)	5768 (82.2)	309 ( 3.6)	1162 (14.2)	733 ( 8.7)	738 ( 9.1)	290 ( 3.6)	1181 (14.1)	165 ( 2.0)	1306 (15.8)	830 ( 9.9)	641 ( 7.8)
Good diet	986 (10.7)	789 (82.4)	39 ( 2.4)	158 (15.2)	116 ( 9.5)	81 ( 8.1)	33 ( 2.2)	164 (15.4)	22 ( 1.1)	175 (16.5)	120 ( 9.8)	77 ( 7.8)
**Physical activity** *												
Inactive	2000 (14.0)	1538 (78.9)	122 ( 4.8)	340 (16.3)	240 ( 9.9)	222 (11.1)	118 ( 5.2)	344 (15.9)	64 ( 2.7)	398 (18.4)	276 (11.9)	186 ( 9.2)
Does not meet guidelines	4087 (41.6)	3203 (80.3)	188 ( 3.8)	696 (15.9)	441 ( 9.5)	443 (10.2)	162 ( 4.0)	722 (15.7)	95 ( 2.0)	789 (17.7)	501 (11.1)	383 ( 8.5)
Meets guidelines	3920 (44.3)	3259 (85.3)	112 ( 2.4)	549 (12.4)	335 ( 7.0)	326 ( 7.7)	95 ( 1.9)	566 (12.9)	60 ( 1.4)	601 (13.4)	359 ( 7.3)	302 ( 7.4)
**C-peptide** *												
Low (<.26 nmol/L)	1353 (15.3)	1269 (95.4)	6 ( 0.2)	78 ( 4.3)	41 ( 2.2)	43 ( 2.4)	9 ( 0.5)	75 ( 4.1)	5 ( 0.2)	79 ( 4.3)	42 ( 2.2)	42 ( 2.3)
Normal (.26–1.03 nmol/L)	6549 (67.3)	5534 (86.6)	57 ( 0.5)	958 (12.9)	433 ( 5.4)	582 ( 8.0)	37 ( 0.3)	978 (13.0)	18 ( 0.2)	997 (13.1)	438 ( 5.4)	577 ( 8.0)
High (>1.03 nmol/L)	2105 (17.4)	1197 (54.0)	359 (17.1)	549 (29.0)	542 (25.9)	366 (20.1)	329 (16.8)	579 (29.3)	196 ( 9.3)	712 (36.7)	656 (32.0)	252 (14.0)	
**Total cholesterol** *												
Good (<200 mg/dL)	4900 (49.9)	4122 (87.0)	134 ( 1.6)	644 (11.4)	358 ( 5.5)	420 ( 7.5)	128 ( 1.6)	650 (11.4)	64 ( 0.8)	714 (12.2)	403 ( 6.1)	375 ( 6.9)
Elevated (200–239 mg/dL)	3167 (31.5)	2444 (79.5)	156 ( 3.9)	567 (16.6)	378 (10.1)	345 (10.3)	142 ( 4.6)	581 (15.9)	83 ( 2.2)	640 (18.2)	427 (11.8)	296 ( 8.6)
High (>=240 mg/dL)	1940 (18.6)	1434 (74.5)	132 ( 6.9)	374 (18.6)	280 (13.6)	226 (12.0)	105 ( 5.3)	401 (20.2)	72 ( 3.7)	434 (21.8)	306 (15.0)	200 (10.6)
**CRP** *												
Normal (0.1- <0.3 mg/dL)	6667 (73.2)	5584 (85.5)	163 ( 1.8)	920 (12.7)	525 ( 6.6)	558 ( 8.0)	145 ( 1.8)	938 (12.8)	85 ( 1.0)	998 (13.5)	573 ( 7.1)	510 ( 7.4)
Mild inflammation (0.3–1 mg/dL)	2497 (20.6)	1793 (73.7)	183 ( 6.8)	521 (19.5)	371 (13.7)	333 (12.6)	162 ( 6.3)	542 (20.0)	92 ( 3.5)	612 (22.8)	423 (15.8)	281 (10.5)
Significant inflammation (>1 mg/dL)	843 ( 6.3)	623 (73.8)	226 (12.0)	468 (29.1)	120 (13.3)	100 (13.0)	68 ( 9.8)	152 (16.4)	42 ( 5.7)	178 (20.5)	140 (17.0)	80 ( 9.2)

* indicates significant difference among NASH, simple, and normal groups identified by each method, at p<0.05.

a indicates significant difference for HAIR score only

**Table 2:** Odds ratio (OR) and adjusted odds ratio (AOR) and 95% confidence interval of the factors associated with NASH as identified through 3 non-invasive methods (reference population of normal-to-mild hepatic steatosis) [N=10,007].

	HAIR		Gholam		NASH liver fat score		All 3 methods		At least 1 method	
	OR	AOR	OR	AOR	OR	AOR	OR	AOR	OR	AOR
**Age**										
35–49 vs 20–34	**2.5 [1.3–4.9]**	1.1 [0.5–2.3]	**1.8 [1.3–2.6]**	1.1 [0.7–1.6]	**2.1 [1.2–3.7]**	0.9 [0.5–1.7]	1.7 [0.8–3.7]	0.6 [0.3–1.4]	**1.8 [1.3–2.6]**	1.1 [0.7–1.6]
50–64 vs 20–34	**5.7 [3.2–10.1]**	1.4 [0.6–3.1]	**3.3 [2.4–4.6]**	1.4 [0.9–2.1]	**3.4 [1.8–6.4]**	0.9 [0.4–1.9]	**3.8 [1.8–8.0]**	0.8 [0.3–2.0]	**3.2 [2.3–4.5]**	1.3 [0.8–1.9]
65+ vs 20–34	**5.6 [2.8–11.0]**	1.0 [0.4–2.4]	**3.0 [2.0–4.4]**	1.0 [0.6–1.7]	**2.6 [1.4–4.9]**	0.5 [0.2–1.1]	**3.8 [1.6–8.7]**	0.6 [0.2–1.6]	**2.9 [2.0–4.2]**	0.9 [0.6–1.5]
**Race/ethnicity**										
Black vs White	1.0 [0.7–1.4]	1.0 [0.7–1.5]	0.9 [0.7–1.1]	0.9 [0.7–1.1]	0.9 [0.6–1.2]	0.8 [0.5–1.2]	0.8 [0.5–1.2]	0.7 [0.5–1.1]	0.9 [0.7–1.1]	0.9 [0.7–1.2]
Mexican-American vs White	**1.8 [1.3–2.6]**	**1.6 [1.0–2.4]**	**1.9 [1.5–2.5]**	**1.7 [1.3–2.2]**	**2.0 [1.3–3.1]**	1.4 [0.8–2.3]	**2.3 [1.4–3.7]**	1.8 [1.0–3.3]	**1.8 [1.5–2.3]**	**1.6 [1.2–2.0]**
Other vs White	1.1 [0.5–2.3]	**3.9 [1.3–11.6]**	1.1 [0.7–1.7]	1.5 [0.8–2.7]	1.7 [0.8–3.4]	**3.9 [1.4–10.9]**	1.7 [0.8–3.8]	**7.2 [1.9–27.6]**	1.2 [0.8–1.8]	1.5 [0.9–2.5]
**Sex**										
female vs male	0.8 [0.6–1.1]	0.8 [0.5–1.1]	**0.6 [0.5–0.7]**	**0.6 [0.4–0.7]**	0.9 [0.6–1.3]	0.9 [0.6–1.5]	0.9 [0.5–1.4]	0.9 [0.5–1.7]	**0.6 [0.5–0.7]**	**0.6 [0.5–0.7]**
**Location**										
rural vs urban	**1.8 [1.3–2.6]**	**1.6 [1.1–2.3]**	1.2 [0.9–1.6]	1.1 [0.8–1.5]	**1.7 [1.1–2.5]**	**1.6 [1.0–2.6]**	**2.0 [1.2–3.2]**	1.7 [1.0–2.9]	1.2 [1.0–1.6]	1.1 [0.8–1.5]
**Education**										
less than high school vs high school	**1.6 [1.2–2.2]**	1.2 [0.8–1.8]	**1.3 [1.1–1.7]**	1.0 [0.8–1.4]	1.2 [0.9–1.6]	0.9 [0.6–1.4]	**2.0 [1.3–3.2]**	1.6 [0.8–2.9]	**1.3 [1.0–1.6]**	1.0 [0.7–1.3]
more than high school vs high school	**0.6 [0.4–0.8]**	0.8 [0.5–1.3]	0.9 [0.7–1.1]	1.0 [0.8–1.4]	**0.6 [0.4–0.8]**	0.8 [0.6–1.2]	0.7 [0.5–1.1]	1.0 [0.6–1.8]	0.8 [0.6–1.0]	1.0 [0.7–1.3]
**Federal income ratio**										
<1 vs >2	1.3 [0.9–1.9]	1.1 [0.7–1.6]	1.2 [0.9–1.6]	1.2 [0.8–1.8]	1.5 [1.0–2.3]	1.1 [0.6–1.9]	1.3 [0.8–2.3]	0.9 [0.5–1.9]	1.2 [0.9–1.6]	1.2 [0.8–1.7]
1–2 vs >2	1.4 [0.9–2.1]	1.1 [0.7–1.7]	1.0 [0.8–1.4]	1.0 [0.7–1.5]	1.2 [0.7–1.9]	1.0 [0.6–1.6]	1.4 [0.9–2.4]	1.0 [0.5–1.9]	1.0 [0.8–1.3]	1.0 [0.7–1.4]
**Where born**										
Mexico vs United States	1.4 [0.9–2.3]	1.3 [0.7–2.5]	**1.7 [1.2–2.3]**	1.0 [0.7–1.4]	**2.0 [1.2–3.3]**	**1.8 [1.0–3.2]**	**1.9 [1.0–3.5]**	1.5 [0.7–3.1]	**1.6 [1.2–2.2]**	1.0 [0.7–1.3]
Other vs United States	**0.3 [0.1–0.7]**	**0.2 [0.0–0.6]**	0.8 [0.6–1.2]	0.7 [0.3–1.4]	0.8 [0.3–1.7]	0.3 [0.1–1.0]	**0.4 [0.1–0.9]**	**0.1 [0.0–0.6]**	0.9 [0.6–1.3]	0.7 [0.4–1.3]
**How long lived in US**										
Less than 5 years vs Entire life	**0.3 [0.1–0.7]**	0.5 [0.1–1.5]	1.1 [0.5–2.6]	1.1 [0.4–3.0]	0.4 [0.2–1.0]	0.3 [0.1–1.1]	0.4 [0.1–1.1]	0.4 [0.1–1.7]	1.0 [0.4–2.3]	1.0 [0.4–2.6]
5–10 years vs Entire life	**0.1 [0.0–0.4]**	**0.2 [0.0–0.7]**	0.5 [0.3–1.0]	0.6 [0.3–1.2]	1.3 [0.3–6.0]	1.7 [0.4–7.4]	**0.1 [0.0–0.5]**	**0.1 [0.0–0.5]**	0.8 [0.3–2.2]	0.9 [0.4–2.1]
More than 10 years vs Entire life	0.7 [0.4–1.2]		1.1 [0.8–1.6]		1.1 [0.6–1.9]		0.9 [0.5–1.7]		1.2 [0.8–1.6]	
**Waist-Hip Ratio**										
risk for women (>= 0.85)/risk for men (>= 0.9) vs healthy	**17.6 [7.0–44.3]**	**4.3 [1.6–11.5]**	**7.3 [5.2–10.1]**	**3.2 [2.2–4.6]**	**11.8 [5.9–23.5]**	**3.4 [1.6–7.2]**	**24.3 [7.8–75.8]**	**7.5 [2.6–21.6]**	**7.2 [5.3–9.8]**	**3.0 [2.1–4.4]**
**Smoking status**										
Current vs Never	**0.4 [0.2–0.7]**	**0.3 [0.1–0.4]**	**0.5 [0.4–0.6]**	**0.4 [0.3–0.5]**	**0.6 [0.3–0.9]**	**0.4 [0.2–0.6]**	**0.3 [0.2–0.7]**	**0.2 [0.1–0.4]**	**0.5 [0.4–0.7]**	**0.4 [0.3–0.5]**
Former vs Never	**2.6 [1.7–3.7]**	1.4 [0.9–2.2]	**1.6 [1.3–1.9]**	1.0 [0.7–1.2]	**2.2 [1.5–3.2]**	1.3 [0.9–2.0]	**2.3 [1.4–3.8]**	1.2 [0.7–2.1]	**1.7 [1.3–2.0]**	1.0 [0.8–1.3]
**Alcohol use**										
Current Drinkers vs Never drinkers	**0.6 [0.4–1.0]**	1. [0.6–2.0]	**0.7 [0.5–1.0]**	0.9 [0.6–1.4]	0.6 [0.3–1.0]	1.1 [0.6–2.2]	0.8 [0.4–1.5]	1.6 [0.7–3.6]	**0.7 [0.5–0.9]**	0.9 [0.6–1.3]
Historical drinkers vs Never drinkers	**1.5 [1.0–2.3]**	1.5 [0.9–2.5]	1.1 [0.8–1.5]	1.1 [0.7–1.6]	1.4 [0.9–2.3]	1.6 [0.9–2.8]	**2.0 [1.0–3.7]**	**2.3 [1.1–4.7]**	1.1 [0.8–1.5]	1.1 [0.7–1.6]
**Healthy eating index**										
poor diet vs good diet	1.2 [0.7–2.0]	1.1 [0.6–2.0]	0.7 [0.5–1.1]	0.7 [0.5–1.2]	1.0 [0.4–2.3]	0.8 [0.3–2.3]	1.3 [0.5–3.4]	1.3 [0.5–3.4]	0.8 [0.5–1.2]	0.8 [0.5–1.2]
needs improvement vs good diet	**1.5 [1.0–2.2]**	1.6 [1.0–2.6]	0.9 [0.7–1.2]	1.0 [0.7–1.3]	1.7 [0.9–3.1]	1.5 [0.6–3.8]	1.8 [0.9–3.5]	1.8 [0.8–4.2]	1.0 [0.8–1.3]	1.0 [0.7–1.4]
**Physical activity**										
Inactive vs Meets guidelines	**2.2 [1.5–3.2]**	1.1 [0.7–1.7]	**1.5 [1.2–1.9]**	1.1 [0.8–1.5]	**3.0 [2.0–4.6]**	1.6 [1.0–2.7]	**2.1 [1.1–4.0]**	1.1 [0.5–2.1]	**1.8 [1.4–2.2]**	1.2 [0.9–1.7]
Does not meet guidelines vs Meets guidelines	**1.7 [1.2–2.4]**	1.2 [0.8–1.7]	**1.4 [1.2–1.8]**	1.2 [0.9–1.5]	**2.3 [1.6–3.2]**	**1.6 [1.1–2.2]**	**1.5 [1.0–2.3]**	1.0 [0.7–1.6]	**1.6 [1.3–2.0]**	**1.3 [1.0–1.6]**
**C-Peptide**										
low (<.26 nmol/L) vs normal (.26–1.03 nmol/L)	0.4 [0.1–1.3]	0.7 [0.2–2.3]	**0.4 [0.2–0.6]**	**0.6 [0.4–0.9]**	1.2 [0.4–3.1]	2.0 [0.8–5.3]	0.9 [0.2–3.5]	1.6 [0.4–6.6]	**0.4 [0.2–0.6]**	**0.6 [0.4–0.9]**
high (>1.03 nmol/L) vs normal (.26–1.03 nmol/L)	**59.1 [34.3–102]**	**35.5 [20.0–63.0]**	**7.7 [5.9–10.0]**	**5.4 [4.1–7.0]**	**77.4 [40.5–148]**	**50.3 [25.2–100]**	**67.3 [28.0–162]**	**40.9 [15.7–106]**	**9.5 [7.4–12.3]**	**6.6 [5.1–8.5]**
**Serum cholesterol**										
elevated (200–239 mg/dL) vs good (<200 mg/dL)	**2.7 [1.7–4.2]**	1.5 [0.9–2.3]	**2.0 [1.6–2.6]**	**1.3 [1.0–1.7]**	**3.2 [2.0–5.1]**	**2.0 [1.3–3.2]**	**3.0 [1.6–5.6]**	1.8 [1.0–3.3]	**2.1 [1.7–2.7]**	**1.4 [1.1–1.8]**
high (>=240 mg/dL) vs good (<200 mg/dL)	**5.0 [3.2–7.9]**	**2.0 [1.2–3.3]**	**2.9 [2.2–3.8]**	**1.5 [1.1–2.0]**	**4.0 [2.4–6.5]**	**1.7 [1.1–2.8]**	**5.3 [2.8–10.2]**	**2.2 [1.1–4.4]**	**2.9 [2.2–3.7]**	**1.4 [1.1–1.9]**
**CRP**										
mild inflammation (0.3–1 mg/dL) vs normal (0.1– <0.3 mg/dL)	**4.3 [2.8–6.5]**	**2.1 [1.4–3.2]**	**2.4 [1.8–3.2]**	**1.6 [1.2–2.2]**	**4.1 [2.9–5.8]**	**2.1 [1.4–3.0]**	**4.1 [2.6–6.5]**	**1.9 [1.2–3.1]**	**2.6 [1.9–3.4]**	**1.7 [1.3–2.2]**
significant inflammation (>1 mg/dL) vs normal (0.1– <0.3 mg/dL)	**5.9 [3.8–9.3]**	**2.0 [1.3–3.3]**	**2.3 [1.8–3.1]**	1.3 [1.0–1.8]	**6.4 [4.3–9.6]**	**2.2 [1.5–3.4]**	**6.7 [4.1–11.0]**	**2.1 [1.2–3.8]**	**2.8 [2.0–3.8]**	**1.5 [1.0–2.0]**

** Bold indicates significance at p<0.05

OR=Odds ratio AOR=Adjusted odds ratio
